# Plasma Exchange for the Recurrence of Primary Focal Segmental Glomerulosclerosis in Adult Renal Transplant Recipients: A Meta-Analysis

**DOI:** 10.1155/2015/639628

**Published:** 2015-11-30

**Authors:** Georgios Vlachopanos, Argyrios Georgalis, Harikleia Gakiopoulou

**Affiliations:** ^1^Department of Nephrology, “Asklepieion” General Hospital, Vas. Pavlou Avenue 1, 16673 Athens, Greece; ^2^Department of Transplantation Immunology and Nephrology, University Hospital Basel, Spitalstrasse 21, 4056 Basel, Switzerland; ^3^1st Department of Pathology, School of Medicine, National and Kapodistrian University of Athens, Mikras Asias Street 75, 11527 Athens, Greece

## Abstract

*Background*. Posttransplant recurrence of primary focal segmental glomerulosclerosis (rFSGS) in the form of massive proteinuria is not uncommon and has detrimental consequences on renal allograft survival. A putative circulating permeability factor has been implicated in the pathogenesis leading to widespread use of plasma exchange (PLEX). We reviewed published studies to assess the role of PLEX on treatment of rFSGS in adults.* Methods*. Eligible manuscripts compared PLEX or variants with conventional care for inducing proteinuria remission (PR) in rFSGS and were identified through MEDLINE and reference lists. Data were abstracted in parallel by two reviewers.* Results*. We detected 6 nonrandomized studies with 117 cases enrolled. In a random effects model, the pooled risk ratio for the composite endpoint of partial or complete PR was 0,38 in favour of PLEX (95% CI: 0,23–0,61). No statistical heterogeneity was observed among included studies (*I* ^2^ = 0%, *p* = 0,42). On average, 9–26 PLEX sessions were performed to achieve PR. Renal allograft loss due to recurrence was lower (range: 0%–67%) in patients treated with PLEX.* Conclusion*. Notwithstanding the inherent limitations of small, observational trials, PLEX appears to be effective for PR in rFSGS. Additional research is needed to further elucidate its optimal use and impact on long-term allograft survival.

## 1. Introduction

Focal segmental glomerulosclerosis is a histologic pattern of glomerular lesion which is associated with distinct clinicopathological entities. These are classified either as primary (idiopathic) or as secondary to a diverse array of causes. Although a variety of involved mechanisms have been proposed in each case, dysregulation of the podocyte is critical for the development of this syndrome and leads to a common pathogenetic pathway in which disruption of the glomerular structure occurs and inexorably progresses to chronic kidney disease. Primary focal segmental glomerulosclerosis is currently the most common primary glomerulopathy leading to end-stage renal disease (ESRD) in the USA [1]. The recurrence of primary focal segmental glomerulosclerosis (rFSGS) after kidney transplantation was reported as early as 1972 [2]. The rate of recurrence in first-renal allografts ranges from 20% to 30% and is correlated with suboptimal allograft survival [3, 4]. Risk factors for rFSGS include early onset (children <15 years) and aggressive course of disease in native kidneys with rapid progression to ESRD and loss of a previous allograft from rFSGS [3, 4]. Recurrence typically presents with heavy proteinuria during the first month after kidney transplantation. Histologic findings are usually unremarkable on light microscopy while diffuse effacement of podocyte foot processes is only documented with electron microscopy. The cause of primary as well as of rFSGS has been suggested to be a circulating 50 kDa plasma protein that targets the podocytes, has a high affinity for galactose, and supposedly forms a complex with immunoglobulins. However, it is debatable if it could be the sole or unique cause of the disease or, even more, merely an epiphenomenon. In support of this controversy, the quest for this protein has proven so far to be elusive. More recently, the soluble urokinase plasminogen activator receptor (suPAR) was also suggested that it may be identified with the humoral permeability factor [5]. However, initial promising results have not been confirmed in subsequent studies and the uncertainty over the permeability factor still persists.

Plasma exchange or its variants like immunoadsorption (PLEX) can theoretically remove the circulating factor since it is bound to immunoglobulins and both have been applied in the treatment of rFSGS. Early case reports and case series reported beneficial results with the use of PLEX for proteinuria remission (PR) in rFSGS as long as it is performed promptly after recognition of recurrence [6, 7]. Equally encouraging results have also been reported in the pediatric population with rFSGS [8]. Relapse can occur during rapid tapering of PLEX sessions and usually requires prolonged reinstitution of PLEX sessions; hence, individualization of treatment schedule depending on clinical response has been elegantly proposed [9]. PLEX has also been applied preoperatively for the prevention of rFSGS but the results are less favourable [10]. Given that small sample size and lack of control group in early studies limit the generalizability of their conclusions, there is a pressing need for an integrated approach to available data about the use of PLEX in this clinical context and for a critical synthesis of the evidence base to produce meaningful conclusions. We systematically reviewed the literature in order to conduct a meta-analysis of published studies to clarify the impact of therapeutic PLEX on PR in adult patients with rFSGS. 

## 2. Subjects and Methods

We searched through the electronic database MEDLINE (US National Library of Medicine, National Institutes of Health) for eligible manuscripts that were published till March 1, 2013. The scan was performed using two different search themes which we combined with the Boolean operator “AND.” The first one used terms referring to the recurrence of primary focal segmental glomerulosclerosis after kidney transplantation, whereas the second one used terms concerning PLEX (e.g., plasmapheresis, immunoadsorption, therapeutic apheresis, cascade, or double plasma filtration). Furthermore, we thoroughly reviewed citations from reference lists of initially retrieved articles to identify additional manuscripts. 

The first two authors (Georgios Vlachopanos and Argyrios Georgalis) independently and in parallel evaluated the abstracts of all detected manuscripts. In the next stage, the full text of eligible studies was critically appraised to exclude uncontrolled case series and to check for satisfaction of inclusion criteria. The latter were considered to be fulfilled if examined studies had compared postoperative treatment with PLEX or variants versus conventional care for PR in adult renal transplant recipients with rFSGS. Any disagreement between the two authors was resolved through discussion under supervision of a senior researcher (Harikleia Gakiopoulou). Extracted information consisted of demographic, clinical, and laboratory data as well as details of the treatment protocol.

The composite of partial or complete PR (as the most relevant surrogate of disease remission) was set as the primary outcome. Although some differences in the definition of PR are evident in literature, we adopt the schema that defines partial remission as >50% reduction in proteinuria from baseline value and ideally to <3,5 g/day and complete remission as proteinuria <0,5 g/day [9]. Estimates of the pooled risk ratio for the study outcome were calculated using the Mantel-Haenszel random effects model with 95% confidence intervals (95% CI). Furthermore, we assessed reported rates of renal allograft loss due to recurrence. We investigated the presence of statistical heterogeneity using* I* ^2^ and Cochran* Q*-test. All statistical analyses were done using Review Manager, version 5.3 (RevMan, computer program, Copenhagen: The Nordic Cochrane Centre, The Cochrane Collaboration, 2014).

## 3. Results and Discussion

**Figure 1:**
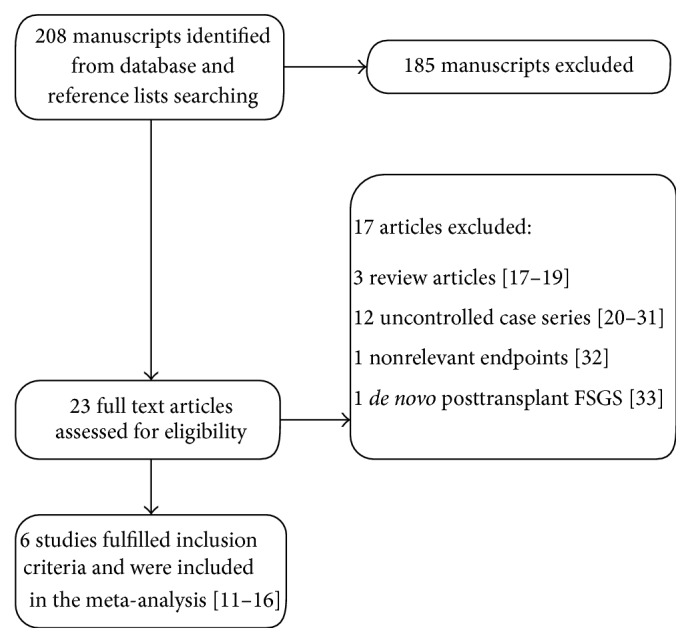
Flow diagram of systematic literature search. FSGS: focal segmental glomerulosclerosis.

Our search strategy yielded a total of 208 unique manuscripts (Figure 1). After title and abstract scanning, 185 manuscripts were excluded because either was totally irrelevant, did not fall within the scope of this meta-analysis (e.g., pediatric population), or reported isolated case reports. The remaining 23 manuscripts underwent further full text review. Three of them were excluded because they were review articles [17–19] and 14 were excluded because they reported uncontrolled case series, had no relevant outcomes, or included cases of* de novo* FSGS after kidney transplantation [20–33]. Out of the 6 studies that ultimately met our inclusion criteria, none was a randomized controlled study and 4 of them used historical control groups [11–16].

A total of 117 patients from these 6 observational studies were included in the analysis, of whom 58 were treated with PLEX and 59 served as controls and received supportive care only. Mean patient age ranged from 21 to 40 years in the PLEX group and from 25 to 33 years in controls (Table 1). Most patients were males; female gender ranged from 20% to 44% in the PLEX group and from 20% to 40% in controls. A living donor was available in 17%–31% of patients in the PLEX group and in 0%–33% of controls, when reported. The vast majority of patients had received their first-renal allograft; 33%–90% in the PLEX group and 79%–100% in controls. Mean proteinuria at recurrence ranged from 5,2 to 12,0 g/day in the PLEX group and from 5,8 to 11,6 g/day in controls. Finally, median time to recurrence was 2–6 days in the PLEX group and 5–81 days in controls.

PLEX prescription varied among the 6 studies reflecting the degree of clinical heterogeneity (Table 2). In four of them classic PLEX was applied whereas in one study the method of double filtration plasmapheresis was used. The usual plasma volume processed was 1,5x individual plasma volume. Mean total number of sessions to achieve PR ranged from 9 to 26. In the study by Canaud et al. [15], a standard immunosuppressive treatment regimen was recommended in cases of rFSGS which consisted of (1) high dose oral steroids (1 mg/kg/day) for the first 4 weeks followed by tapering, (2) intravenous cyclosporine (2 mg/kg, targeting C0 trough blood levels: 200–400 ng/mL) for 2 weeks followed by oral treatment (targeting C2 peak blood levels: 1.200–1.400 ng/mL), and (3) mycophenolate mofetil 2.000 mg bid. It is of note that in the same study some patients in the historical controls group were also treated with PLEX. However, we decided to include this study in the meta-analysis because PLEX treatment was not standardized in historical controls whereas in the active treatment arm an intensive and prolonged course of PLEX was employed as part of an overall treatment plan. Similarly, in Otsubo et al. [13] some patients in the PLEX group were treated with preemptive PLEX before kidney transplantation. We also decided to include this study in the meta-analysis because pretransplant PLEX did not prevent disease recurrence in these patients and, therefore, we believe that it did not influence response to posttransplant PLEX. Data on angiotensin converting enzyme (ACE) inhibitors, angiotensin receptor blockers (ARBs), and rituximab use among PLEX patients and controls were generally underreported and inconsistent.

**Figure 2:**
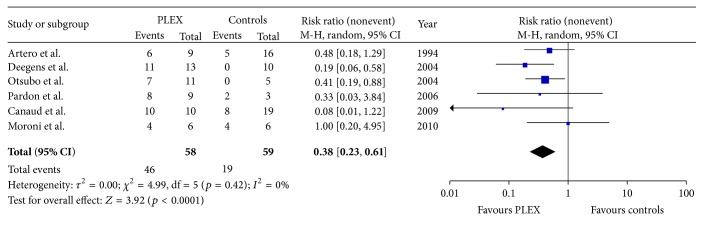
Forest plot of the effect of plasma exchange on the composite endpoint of partial or complete proteinuria remission. CI: confidence intervals; M-H: Mantel-Haenszel method; PLEX: plasma exchange.

All 6 studies were included in the quantitative meta-analysis (Figure 2). Using the Mantel-Haenszel random effects model, the pooled risk ratio for the composite endpoint of partial or complete PR was 0,38 for patients treated with PLEX compared with controls (95% CI: 0,23–0,61). This result was primarily driven by the studies of Deegens et al. [12] and Otsubo et al. [13], in which a statistically significant benefit for patients treated with PLEX was demonstrated (risk ratio: 0,19, 95% CI: 0,06–0,58 and risk ratio: 0,41, 95% CI: 0,19–0,88, resp.). In Moroni et al. [16], there was no difference between the two groups (risk ratio: 1,00, 95% CI: 0,20–4,95), whereas in the rest three studies there was a trend in favour of PLEX but it did not reach statistical significance. Some minor discrepancies in the definition of PR existed among included studies; nevertheless, after careful inspection of each article results, there was not any disagreement between reviewers on the inclusion of PR cases as reported by the studies' authors. The statistical heterogeneity among included studies was negligible (*I* ^2^ = 0%, *Q*-test *p* = 0,42).

Renal allograft loss due to recurrence was lower in the PLEX group, varying from 0% to 67% over a follow-up time that ranged from 16 to 73 months (Table 3). In contrast, renal allograft loss due to recurrence was between 33% and 100% in controls over a follow-up time of 43 to 83 months. However, lack of reporting individual patient data in some studies and allograft survival at a specific time-point (e.g., 1-year or 3-year allograft survival) precluded an accurate quantitative comparison of allograft survival between the two groups in the form of a forest plot. PLEX is an interventional procedure whose most common complications include hypotension, anaphylactoid reactions, and hypocalcemia. Without ignoring the fact that a small number of cases were enrolled, it is remarkable that no such complications were reported in the included studies highlighting PLEX safety in rFSGS treatment.

Determinants of the response to treatment appear to be the benign histologic findings and the timely institution of PLEX. These were already identified in the chronologically first included study by Artero et al. [11]. The absence of glomerular lesions on light microscopy and the presence of only podocyte foot process effacement on electron microscopy represent an early disease stage when no irreversible tissue damage has occurred. Complete resolution of podocyte foot process effacement has been demonstrated after successful treatment and induction of clinical remission. On the other hand, the development of typical segmental sclerotic lesions in renal allograft biopsy denotes disease progression to a later stage characterized also by interstitial fibrosis and has been associated with poor outcomes. Observed response to rapid institution of PLEX can also be explained by this very concept of disease staging and of reversibility of early histologic lesions. It is generally recommended that PLEX should be initiated immediately after diagnosis of recurrence or, at least, within the first 14 days; beyond that time-point, PLEX treatment has often proved to be unsuccessful. The present meta-analysis focuses on the outcome of complete or partial PR after initial PLEX therapy. However, it is possible that rFSGS relapses upon stopping or tapering of PLEX. A second or third PLEX course may be useful in this clinical scenario. For PLEX-dependent patients, treatment may have to be performed for prolonged periods, even years in rare cases.

The risk of bias in nonrandomized studies is naturally higher than in randomized ones and the studies included in the present meta-analysis could not be an exception to this rule. Study quality can be considered rather low; methods to address confounding were not generally applied. The sample size of all of them was small. As a result, calculation of a meta-analysis forest plot was only feasible for the total of enrolled cases and no further subgroup sensitivity analyses could be performed. Effect estimates were crude and were not adjusted for potential confounding factors. Furthermore, uncertainty over the results is substantial as the wide confidence intervals hint. The possibility for a positive publication bias is increased with small scale studies but since included studies were few, a formal assessment for publication bias in the form of a funnel plot was not performed. Although statistical heterogeneity was negligible, this finding should be interpreted with caution, because clinical heterogeneity was apparent among included studies. Several differences in the treatment protocol existed, such as in the frequency and duration of PLEX treatment, which varied considerably, as well as in the plasma volume processed in each PLEX session. Besides that, active treatment and control groups were not always treated equally. This can be best exemplified by the fact that the use of ACE inhibitors or ARBs was not applied evenly in all cases of the two groups; it was more common for patients belonging to historical control groups not being treated with ACE inhibitors or ARBs.

Established or novel medications that have been used in conjunction with PLEX for treatment of rFSGS include calcineurin inhibitors (mainly cyclosporin), rituximab, and costimulation blockers (abatacept and belatacept). Although the standard oral doses of cyclosporine, which are used for prevention of acute rejection, have not had an effect in rFSGS, the use of higher intravenous doses has been associated with PR [15, 34]. This is attributed in part to the direct effect of cyclosporine to the podocyte cytoskeleton, while the attainment of higher blood levels is thought to counterbalance the increased serum cholesterol observed in nephrotic patients with rFSGS [35]. Rituximab is a chimeric monoclonal antibody against the CD20 antigen of B-lymphocytes. Complete remission of rFSGS was reported in a young renal transplant recipient who received rituximab for treatment of posttransplant lymphoproliferative disorder [36]. Along with that observation, several reports of rituximab administration for rFSGS emerged and a systematic review of 39 rFSGS cases treated with rituximab demonstrated partial or complete response to therapy in 64% (25/39) of them [37]. Abatacept and belatacept are fusion proteins that target the B7-1(CD80) and B7-2(CD86) molecules inhibiting costimulation and subsequent activation of T-lymphocytes. Yu et al. reported that abatacept administration induced partial or complete remission in four patients with resistant rFSGS [38]. However, these initial promising results of costimulation blockade have not been confirmed in abatacept and belatacept studies that followed [39, 40].

## 4. Conclusion

This meta-analysis is the first attempt to synthesize the results of 6 nonrandomized studies including 117 renal transplant recipients and provides relatively valid evidence that implementation of PLEX for rFSGS leads to disease remission and potentially protects from renal allograft loss. rFSGS is a dreaded complication of kidney transplantation with an incompletely understood pathogenesis. Treatment with PLEX is etiologic and targets the removal of the harmful circulating permeability factor. It should be part of a complete management plan that incorporates the administration of an ACE inhibitor or ARB, a calcineurin inhibitor, and possibly rituximab. Components of PLEX treatment such as duration and frequency are not yet unanimously agreed. Thus, further large scale studies with longer follow-up are required to determine the optimal use of PLEX in the clinical setting of rFSGS.

## Figures and Tables

**Table 1:** Characteristics of cases in included studies. Ctr: control group; NR: not reported; PLEX: plasma exchange group; Tx: transplantation.

Study	Cases		Mean age at Tx (years)		Female gender (%)		Living donor Tx (%)		1st graft (%)		Mean proteinuria at recurrence (g/day)		Median time to recurrence (days)	
	PLEX	Ctr	PLEX	Ctr	PLEX	Ctr	PLEX	Ctr	PLEX	Ctr	PLEX	Ctr	PLEX	Ctr
Artero et al. (1994) [11]	9	16	30	30	44	31	NR	NR	NR	NR	12,0	11,6	NR	NR

Deegens et al. (2004) [12]	13	10	40	33	38	40	31	0	77	100	5,7	5,8	4	9

Otsubo et al. (2004) [13]	11	5	21	25	36	20	NR	NR	NR	NR	NR	NR	3	81

Pardon et al. (2006) [14]	9	3	NR	NR	NR	NR	NR	NR	NR	NR	10,4	8,3	NR	NR

Canaud et al. (2009) [15]	10	19	NR	NR	20	37	20	21	90	79	12,0	NR	2	NR

Moroni et al. (2010) [16]	6	6	NR	NR	0	33	17	33	33	83	5,2	5,8	6	5

**Table 2:** Characteristics of treatment in included studies. DFFP: double filtration plasmapheresis; NR: not reported; PLEX: plasma exchange.

Study	Method	Treatment protocol	Mean total sessions	Plasma volume processed	Mean treatment length (months)
Artero et al. (1994) [11]	PLEX	Daily sessions for 3 consecutive days, then every other day for a total of nine sessions	9	1,5x plasma volume	NR

Deegens et al. (2004) [12]	PLEX	Daily sessions for 3 days, then according to clinical response	24	1,5x plasma volume	12

Otsubo et al. (2004) [13]	DFFP	3 sessions every other day, then according to clinical response	10	NR	NR

Pardon et al. (2006) [14]	NR	NR	16	NR	NR

Canaud et al. (2009) [15]	PLEX	3 sessions per week during 3 weeks, followed by 2 sessions per week during 3 weeks, 1 session per week until month 3, 2 sessions per month until month 5, and finally once a month until month 9	26	NR	10

Moroni et al. (2010) [16]	PLEX	3 sessions per week during the first 3 weeks, followed by 2 sessions per week during 3 weeks and then tailored to clinical response	NR	1,0x plasma volume	14

**Table 3:** Renal allograft loss due to recurrence and mean follow-up time. NR: not reported; PLEX: plasma exchange.

Study	PLEX group *n*/*N* (%)	Mean follow-up time (months)	Control group *n*/*N* (%)	Mean follow-up time (months)
Artero et al. (1994) [11]	1/9 (11)	17	8/16 (50)	NR
Deegens et al. (2004) [12]	0/13 (0)	41	5/10 (50)	43
Otsubo et al. (2004) [13]	4/11 (36)	46	5/5 (100)	44
Pardon et al. (2006) [14]	6/9 (67)	NR	1/3 (33)	NR
Canaud et al. (2009) [15]	0/10 (0)	16	10/19 (53)	46
Moroni et al. (2010) [16]	3/6 (50)	73	4/6 (67)	83
